# Impact of COVID-19 Confinement on Adolescent Patients with Anorexia Nervosa: A Qualitative Interview Study Involving Adolescents and Parents

**DOI:** 10.3390/ijerph18084251

**Published:** 2021-04-16

**Authors:** Michael Zeiler, Tanja Wittek, Leonie Kahlenberg, Eva-Maria Gröbner, Martina Nitsch, Gudrun Wagner, Stefanie Truttmann, Helene Krauss, Karin Waldherr, Andreas Karwautz

**Affiliations:** 1 Eating Disorder Unit, Department of Child and Adolescent Psychiatry, Medical University of Vienna, 1090 Vienna, Austria; tanja.wittek@meduniwien.ac.at (T.W.); leonie.kahlenberg@meduniwien.ac.at (L.K.); eva-maria.groebner@meduniwien.ac.at (E.-M.G.); gudrun.wagner@meduniwien.ac.at (G.W.); stefanie.truttmann@meduniwien.ac.at (S.T.); leni.krauss@hotmail.com (H.K.); andreas.karwautz@meduniwien.ac.at (A.K.); 2 Department for Research and Development, Ferdinand Porsche FernFH-Distance Learning University of Applied Sciences, 2700 Wiener Neustadt, Austria; martina.nitsch@fernfh.ac.at (M.N.); karin.waldherr@fernfh.ac.at (K.W.)

**Keywords:** COVID-19, confinement, anorexia nervosa, adolescents, parents, qualitative study, psychological impact

## Abstract

COVID-19-related restrictions may have a serious impact on patients with eating disorders. We conducted semistructured interviews with female adolescent patients with anorexia nervosa (AN) (*n* = 13, 13–18 years) currently receiving inpatient or outpatient treatment and their parents (*n* = 10). We asked for their experiences during COVID-19 confinement regarding everyday life, AN symptoms, and treatment. We used thematic analysis to interpret the data. The main themes identified from the patients’ interviews involved restrictions of personal freedom (i.e., leading to tension between patients and family members, reduced motivation to work on recovery), interruption of the treatment routine (emerging risks through self-monitored weight, challenges/opportunities of teletherapy), changes in AN symptoms (more exposure to triggering situations), COVID-19-related fears, and compulsions but also potential opportunities (less stress, better family relationships). The parents discussed changes in daily routines as negative (challenges in maintaining day structures) and positive (more family time, “slowing down”). They expressed reservations about reduced outpatient monitoring and increased teletherapy and discussed challenges in keeping contact with the child and clinicians during inpatient treatment. Moreover, the parents discussed deteriorations and improvements in the patients’ psychopathology. Clinical implications from these in-depth insights include the importance of strengthening communication between changing staff cohorts, patients, and parents; motivational work; and joint weight monitoring with the therapist.

## 1. Introduction

Emerging evidence suggests that the coronavirus disease 2019 (COVID-19) pandemic has a significant impact on the mental health of the entire population, with individuals suffering from psychiatric disorders, such as eating disorders, being a particular vulnerable group [1,2,3]. Several studies that primarily involved adult patients with anorexia nervosa (AN) and bulimia nervosa (BN) in outpatient treatment indicate that eating disorder symptoms increased during the COVID-19 confinement. This includes increased restrictive eating and binge eating episodes, self-induced vomiting, shape and weight concerns, rumination, and increased drive for exercise and actual physical activity [4,5,6,7,8]. Furthermore, increased levels of anxiety and depression and reduced quality of life were reported [4,5,6]. A longitudinal study in adult AN and BN patients indicates that the recovery process was interrupted during the lockdown, and remitted patients showed a re-exacerbation of symptoms [9]. Moreover, there is evidence that hospital admissions of children and adolescents due to AN symptomatology increased during the COVID-19 confinement [10].

Potential pathways leading to this aggravation of eating disorder symptoms include increased social isolation and less distractions, increased exposure to stressful messages (e.g., via social media), food insecurity, less opportunity to exercise, and limited access to professional health care [7,10,11,12,13,14,15]. During the COVID-19 confinement, eating disorder treatment (medical and weight monitoring and psychotherapy) has shifted to teletherapy/telemedicine in order to guarantee the continuity of care [16,17]. However, there is evidence that the decrease in face-to-face psychotherapy was not accompanied by a corresponding increase in online therapy, indicating that a certain proportion of (at least adult) eating disorder patients did not receive adequate care during this time [5,6]. Although online therapy seems to be well accepted among eating disorder patients [18], particularly in adolescents [11], mental health experts point to potential pitfalls of teletherapy. For example, less sense of privacy and confidentiality when being at home, problems with Internet speed/connection, difficulties regarding building therapeutic alliance, and the feeling that therapeutic online sessions are more “superficial” compared with face-to-face sessions were mentioned [11,19,20,21]. Patients with AN reported the greatest dissatisfaction with online therapy compared with patients with other eating disorder diagnoses; however, the reason for this is unclear [22]. This may have additionally contributed to the exacerbation of eating disorder symptoms during this time. Furthermore, Taylor, Fitzsimmons-Craft, and Graham [23] emphasize that therapists may not be familiar with the use of teletherapeutic approaches, and thus, therapist training is needed.

Besides a negative impact of the COVID-19 confinement on adult eating disorder patients, also positive consequences have been reported in the literature, including greater connection with the family, more time for self-care, taking over responsibility for recovery, increased self-efficacy, and developing alternative coping strategies and resources facilitating recovery [4,5,6,24]. However, it is unclear whether adolescents with eating disorders also experience similar positive consequences.

To date, research on the impact of COVID-19 measures on adolescent patients with eating disorders is scarce. However, it might be that the effects of COVID-19 measures reported on adult patients also apply—to some extent—to adolescent patients. Furthermore, research in general population samples of adolescents indicates that symptoms of depression and anxiety are on the rise [25,26], which are often present in adolescent patients with eating disorders as comorbid conditions. Another study points to an increase in body dissatisfaction and drive for thinness in the general population of adolescents during the pandemic period, which is attributed to the increase in the use of social media and, therefore, to an increase in potentially triggering messages [27]. This could be even more relevant for adolescents diagnosed with eating disorders.

Caregivers often play a key role in the treatment and recovery process of patients with eating disorders; however, the impact of the COVID-19 confinement on caregivers and their role in supporting their loved ones with an eating disorder has not gained much attention so far. A qualitative study involving caregivers of adult patients with AN showed that concerns about the provision of professional health care for patients, managing patients’ and families’ needs, and coping with patients’ aggravating eating disorder symptoms were most challenging for them [24]. Moreover, the challenge to maintain daily routines [28] and increased levels of caregivers’ anxiety and depression were discussed [29]. During the COVID-19 pandemic, parents generally reported higher levels of stress as they had to cope with multiple demands and challenges (e.g., homeschooling of their children, managing their own jobs, job insecurity, financial loss) [30]. For caregivers of adolescents with AN, higher levels of stress might increase expressed emotion, which has been linked to poorer treatment outcomes for adolescent patients [31].

On 16 March 2020, the Austrian government announced restrictive measures due to the COVID-19 pandemic, including closures of school and universities, closure of shops not relevant for basic supply, and curfews. Restrictions ended on 30 April 2020. Additional measures were taken with regard to inpatient eating disorder treatment at the Department of Child and Adolescent Psychiatry, Medical University of Vienna, Austria, where this study was conducted. These include splitting hospital personnel into two cohorts who work alternately in a 2-week rhythm and restrictions for visitors of inpatient adolescents (visitation was only allowed for one parent within a 2-week period). Additionally, inpatients were confined to hospital grounds. Furthermore, face-to-face medical and psychotherapeutic eating disorder treatment for outpatients was reduced to a minimum, and remote interventions (via telephone or video tools, such as “Instahelp”) were offered whenever possible.

Current research indicates that COVID-19-related restrictions may have a serious impact on patients with eating disorders. However, little is known about the experiences of adolescent patients and their parents as well as how adolescents in inpatient treatment experienced COVID-19 restrictions. Moreover, the impact of confinement on eating disorder patients may vary based on the specific measures taken in countries and hospitals, which demonstrates the need for local and setting-based studies. Thus, this qualitative interview study aims to examine the impact of COVID-19 confinement on adolescent patients with AN and their parents in Vienna and its surrounding area with regard to eating disorder symptoms, inpatient and outpatient eating disorder treatment, and daily routines, including family and school life. In this study, we focused on patients with AN for the following reasons: First, our clinic is specialized on the treatment of adolescent AN, and thus, relatively more patients with AN are assigned to our clinic than patients with other types of eating disorders. Second, due to certain personality traits often seen in patients with AN (e.g., rigidity, perfectionism), AN patients might be especially prone to loss of daily structures. Moreover, a previous study indicated that AN patients were less satisfied with remote interventions compared with patients with bulimia nervosa and binge eating disorder. Considering the often life-threatening course of AN, we prioritized our research on this group of patients.

## 2. Materials and Methods

### 2.1. Participants and Procedure

Participants were purposively recruited from the Eating Disorder Unit of the Department of Child and Adolescent Psychiatry, Medical University of Vienna, Austria. We approached adolescent patients aged between 13 and 18 years who were diagnosed with AN according to DSM-5 diagnostic criteria (restrictive and binge/binge–purging subtype, including acutely ill and weight-restored patients) and received inpatient or outpatient treatment. In detail, we approached all inpatients with AN who were admitted to our ward between end of April and May 2020 and who were able to participate in an interview (no organic brain disease, sufficient knowledge of the German language). Outpatients were recruited via an ongoing project evaluating an outpatient cognitive behavioral treatment approach called MANTR-a (Maudsley model of anorexia nervosa treatment for adolescents and young adults) [32] and via psychiatrists/psychotherapists at our clinic. They were consecutively recruited until data saturation was reached (see below). In addition to the patients, we also asked one of their parents to participate in the study. Single semistructured interviews were conducted between end of April and beginning of June 2020 and were held either in person (at the clinic) or via the videoconference tool “Webex” [33], depending on COVID-19-related restrictions (e.g., restricted access to the hospital building for outpatients and parents) and hospital routines (e.g., no option for inpatients to participate in interview via videoconference). The participants were informed about the study goals (“to find out how the COVID-19 pandemic affects the lives of young people with eating disorders and their parents”), and written informed consent was obtained from the patients and parents. Ethical approval for this study was granted by the Ethics Committee of the Medical University of Vienna (#2005/2017).

The sample size for this study was derived based on data saturation, which we defined as the point whereby further data collection would yield no further major themes. This point was determined by mutual agreement of the interviewers. In total, interviews were conducted with 13 female adolescent patients with a mean age of 15.9 years (SD = 1.4). Two approached patients declined to participate without giving specific reasons. Nine patients were diagnosed with AN restrictive type and four patients with AN binge–purging type. At the time of data collection, eight patients received outpatient treatment, and five patients received inpatient treatment (at least for 2 months). Outpatient treatment included individual psychotherapy (cognitive behavioral therapy) as well as weekly or biweekly medical checks by a child and adolescent psychiatrist.

The mean body mass index (BMI) was 16.7 (SD = 1.4), ranging from 13.7 to 19.3. Sex- and age-specific BMI percentile ranged between <1st and 25th percentile (mean = 7.30, SD = 7.71). The mean duration of the eating disorder was 23 months (SD = 16) ranging from 5 to 48 months. Psychiatric comorbidities were diagnosed in four patients (depressive disorder, obsessive–compulsive disorder, or borderline personality trait). Eleven patients lived with both biological parents, and two patients lived with single parents (mothers). Eleven patients had at least one sibling living in the same household.

Additionally, 10 out of the 13 parents (8 mothers, 2 fathers) agreed to participate in the qualitative interview. Their mean age was 49.7 years (SD = 2.4). Seven parents had a university degree, two had an A-level degree, and one had a below A-level degree. Five parents were full-time, and 5 were part-time employed; however, 11 parents reported that the COVID-19 confinement has changed their working life (working from home, reduced working time with reduced income). Of the parents who declined participation, two stated to have no time, and one expressed no interest in participating in the study.

No participant (neither patients nor parents) reported a previous infection with COVID-19; two participants reported COVID-19 infections of relatives not living in the same household.

### 2.2. Data Collection and Interview Topic Guide

The topic guide of the semistructured interview (provided in the online Supplementary Material S1) was jointly developed by the research team and discussed with the interviewers. Core topics addressed were general experiences during the time of COVID-19 confinement, family life, school life, course of the eating disorder, and experiences of the eating disorder treatment during this time. The topic guides for the patients’ and parents’ interviews were equivalent.

Interviewers were psychologists and child and adolescent psychiatrists (three female, one male) experienced in the field of adolescent eating disorders (M.Z., T.W., L.K., E.-M.G). Two interviewers (those who performed 17 out of 23 interviews) already had experience with conducting semistructured interviews. All the interviewers participated in an interviewer training given by M.N. With the exception of one interview, the interviewers had no or minimal relationship with the interviewees prior to this study.

Six patient interviews were conducted in person and seven patient interviews via the online meeting software Webex^®^ [33] (five interviewees participated via audio and video, and two interviewees participated via audio only). Interviews with the parents were conducted via “Webex” (total of nine, seven participated via audio and video, and two participated via audio only), except one parent, who was interviewed in person. Generally, we aimed to conduct the online interviews with active video connection on both sides. Due to low Internet speed on the interviewees’ side, the video had to be turned off in some cases. Except the interviewer and interviewee, no other person was present during the interview, facilitating a confidential atmosphere. All the interviews were audiotaped. The interviewers took notes on any salient events/nonverbal signals prior to the start of, during, and after the interview. The duration of the interviews with the patients was between 17 and 44 min (mean: 31.5, SD: 8.0) and between 22 and 51 min (mean: 34.9, SD: 10.2) with the parents.

### 2.3. Data Analysis and Thematic Coding

The interviews were transcribed verbatim. Subsequently, the transcripts were organized in NVIVO 12 Pro software (QSR International, Burlington, MA, USA). We used a thematic analysis approach to analyze the data following the principles of Braun and Clarke [34]. In detail, the coding and analysis procedure was as follows: First, two coders (M.Z., T.W.) familiarized themselves with the text by independently reading through all the transcripts and generating first ideas on potential themes. Then, they coded five interviews together to gain a common understanding of the coding process. The remaining interviews were coded by one of the coders and cross-checked by the second coder afterwards. In a next step, four discussion sessions between the two coders took place to inductively identify themes and subthemes from the data material. The generated themes were checked back against the original data, checked against each other, and iteratively refined until consensus was reached. This process was performed separately for the patients’ and parents’ interviews. Afterwards, the thematic structure was discussed with the broader research team (M.N., K.W.), checked for consistency, and refined afterwards. A description for each theme and subtheme was formulated, and illustrative quotes were selected and translated into English (translated by one author and checked by another one). While translating the quotes, we tried to stay as close to the original wording and sentence structure as possible. The label placed after each quote (e.g., #02) refers to the patient’s and parent’s IDs with “#02” meaning the second patient included in the study or the corresponding parent.

## 3. Results

An overview of the main and subthemes identified from the patients’ and parents’ interviews is provided in Table 1.

### 3.1. Themes Identified from the Patients’ Interviews

Four main themes were identified from the patients’ interviews, which are described below.

#### 3.1.1. A. Restrictions of Personal Freedom

COVID-19-related confinement was perceived as restriction of personal freedom, which has manifested itself in the hospital setting (due to visitor restrictions, restrictions to leave the hospital area), family setting (due to restrictions to leave the living space), and school/leisure setting (due to restrictions to visit school, pursue hobbies, and meet friends). The subthemes include feelings and perceived consequences related to these restrictions.

(A1) *Feeling of being imprisoned and bored*: Patients described feelings of being imprisoned and bored due to lack of the possibility to go to school and pursue hobbies: *“This was like, my God, I′m locked up at home now, and I’m sitting around totally unproductive”* (#02). Inpatients emphasized that “*it is very bad to be in the ward the whole time, not being able to go out, not being able to go home, and being actually locked up for 24 h,”* leading to a *“cabin fever”* (#04).
(A2) *Tensions between patients and family members*: Being with other patients or family members 24/7 favored negative feelings like irritability. *“I notice that the tension between the patients is getting worse. You realize that this closeness is simply unbelievably stressful all the time. You′re stuck together the whole time. The girls are already starting to argue”* (#04). *“And just being at home with your family 24/7 is pretty hard for me, because then it comes to arguments and you get on each other′s nerves”* (#01).
(A3) *Less motivation to work on recovery:* Due to COVID-19 restrictions, the motivation to work on recovery decreased. Personal goals that patients were looking forward to and motivated them to work on recovery (such as vacation with the family and home visits for inpatients when weekly weight goals were achieved) were dropped, which impeded their motivation to gain weight. *”You can’t have that experience of how it feels once you haven’t tried hard enough to reach your weekly goal, or how it feels just to be able to get out once you’ve managed to do it”* (#03).
(A4) *Missing close others:* Due to the visiting regulations in the hospitals and general COVID-19 measures, close others were missed. Inpatients did not see parents, siblings, grandparents, and close friends for a longer period of time, which was experienced as a burden despite the possibility to keep contact via social media and telephone. Furthermore, having to decide who was allowed to visit the patient in the hospital and who was not was experienced as difficult and stressful: *“That is really extremely difficult. It′s actually quite a burden, a really big burden, because you have to think about it for quite a long time: Do I want to see my dad for a week and then my mom, or do I want to see my dad for two weeks and then my mom?”* (#04).

#### 3.1.2. B. Interruption of the Treatment Routine

This theme covers risks, challenges, and opportunities that are associated with changes in the treatment routine, both in the outpatient setting (telemedicine, teletherapy) and in the inpatient setting (change of staff cohorts and treatment offers). The following three subthemes were identified:

(B1) *Risks through self-monitored weight*: Reduced outpatient face-to-face contacts and increased remote interventions caused weight control to be carried out by the patients themselves or by their parents. This was experienced as uncomfortable and *“very stressful because I’m afraid that it won’t be okay”* (#10). These feelings were partly reinforced by not being able to *“debrief this with the doctor”* (#12). Patients reported that self-monitored weight triggered eating disorder-related cognitions. Weight control by parents was further experienced as a limitation of personal autonomy. Moreover, patients reported hiding weight loss from their psychiatrists and parents: *“It went so far that I cheated my mom when weighing by simply drinking a lot of water beforehand, partly because she was terribly worried, and I didn′t want her to worry so much […] But this whole exceptional situation with Corona has also somehow provoked this, because otherwise it would never have come so far. Otherwise, I would have continued to have medical checks in the hospital”* (#12).
(B2) *Challenges and opportunities of teletherapy:* In general, the psychological/psychotherapeutic online treatment was perceived as *“rather satisfactory”* (#07) by the patients whereby a bilateral video connection was preferred over audio connection only because eye contact, facial expressions, and gestures were regarded as important during the therapeutic process. However, face-to-face therapy was preferred in general as difficult topics stay in the doctor’s office: *“For me, it was like, I go to my therapist, I leave my problems there, I go home. […] Now, many things take place in my room, and I have all these problems, these conversations right on the spot. This felt strange for me at the beginning.”* (#09). While receiving therapy without having to travel was seen as advantage of teletherapy, privacy concerns when psychotherapy took place in one’s own room were discussed as main challenge: *“I can′t really tell everything and be 100% honest, because I′m always afraid that someone is listening”* (#01).
(B3) *Changes of staff cohorts and treatment offers in the inpatient setting:* Due to the frequently changing staff cohorts in the psychiatry wards, it was perceived as difficult to build relationships with clinicians, which was additionally aggravated by wearing face masks. It was *“difficult, because we could never concentrate on one person [doctor], who guides us through our whole inpatient stay, but we always had to switch, and that was a bit tedious”* (#05). Furthermore, inpatients partly experienced insufficient agreements between staff from different cohorts. On the positive side, new therapists joined the team and offered new options for treatment and free-time activities like *“hypnosis or yoga, which was somehow uncommon, but also pretty cool”* (#03).

#### 3.1.3. C. Changes in the Eating Disorder and Other Psychopathology

This overarching theme describes deteriorations and improvements in eating disorder symptoms and other psychopathology, which are related to COVID-19 measures and characteristics of a normal course of therapy. The following three subthemes were identified:

(C1) *Boredom and feeling of being observed, triggering eating disorder symptoms:* Feeling bored and isolated at home, experiencing little distraction, and feeling observed by family members during eating and exercising triggered AN-related cognitions, restrictive eating behaviors (e.g., counting calories), obsessive exercising, and binge–purging behavior: *“She [my mother] is at home most of the time, my siblings too. Sometimes, I feel a bit observed, and then I feel bad when I’m eating so much”* (#01). *“I not only control my weight to the gram, but also my food intake. I′ve started to weigh food again, to count calories, and to track steps. […] This was because I′m always at home now. I′m not allowed to go to school because of my blood values […]. I spent lots of time in my “anorectic world”* (#12). AN-related cognitions and behaviors were additionally boosted by social comparisons and exchange with peers: *“Really every one of my classmates posted something like ‘Shit, quarantine, I’m getting fat now.’ And that triggered me so much”* (#13).
(C2) *COVID-19-related fears and compulsions:* This included worries regarding continuation of psychotherapy and medical checks, continuation of school lessons, professional and financial situation of the parents, and fear of a COVID-19 infection and infection of close others, particularly grandparents: *“What I was very worried about was my grandparents […]; my grandma has cancer and is getting chemotherapy, and her immune system is very weak. And my biggest concern was that she will get infected”* (#09). Furthermore, fear of not being able to buy certain foods and increased obsessional washing were discussed.
(C3) *Improvement in symptoms, indicating a normal treatment course:* Other patients reported no direct effects of the COVID-19 pandemic on eating disorder symptoms or exercise behavior but mentioned general improvements in symptoms (e.g., less binge–purging episodes, restrictive eating, AN-related cognitions, and lower levels of depression), indicating a normal treatment course: *“I notice that it is getting better now, that I don’t think about food so much anymore, that after the meal I no longer have this mind spinning […] and yes, therapy helps me a lot”* (#08).

#### 3.1.4. D. Opportunities of the COVID-19 Period

This theme described how the COVID-19 period opened up new opportunities to take care of one’s own needs and promote family cohesion.

(D1) *Less stress allows to pay more attention to own needs:* The patients highlighted that less stress regarding school, leisure, and social contacts allowed to pay more attention to one’s own needs. This has allowed them to better enjoy the time available and to follow their own needs: *“I have escaped from this school stress a little bit and was able to better listen to myself [...] Maybe this was also a step that was necessary so that I could calm down myself a bit”* (#11). Another patient told, *“So I have paid more attention to my emotions and to my needs, what I wanted to do now, and not whether it fits into my schedule. So, I just have done what I really wanted to do and haven’t done things that I didn′t want to do”* (#10). Although having more time for oneself bears the risk to get caught in AN-related cognitions, it also opened up the opportunity to *“work towards the opposite direction”* (#12), towards recovery. Experiencing lower stress levels was further associated with more flexible eating (e.g., trying out new foods) and improved mood.
(D2) *Experiencing a more intensive time with the family:* As many parents were working from home and siblings were at home due to homeschooling, there was more time for joint family activities and conversations, which facilitated growing together as a family. One patient described a barbecue event as follows: *“We were all together, and it was a bit like in a storybook because we often don’t have this family idyll because I don’t eat with them most of the time”* (#09). Another patient told that she has start talking with her sister about her eating disorder during this time: *“Two or three days ago, it happened for the first time since I had this disease that I talked to my sister a little bit about it, and that was very relieving”* (#02). Moreover, visits from parents at the ward were experienced *“more consciously”* (#03) (e.g., because the patient had exclusive time with them);*“I could use the time with my mom more intensively because I spent my time alone with her every day*” #08).
(D3) *Promoting autonomy and self-organization skills:* Finally, patients acknowledged that the interruption in everyday and school routines enabled the promotion of autonomy and self-organization skills, including working autonomously on school assignments and planning of the day structure. For example, a patient positively highlighted *“that I am a bit more on my own, that I have to look after myself, that I have to create a daily routine for myself, that the school doesn′t do that for me, that the parents can′t always do that for me”* (#13).

### 3.2. Themes Identified from the Parents’ Interviews

Three main themes were identified from the patients’ interviews, which are described below.

#### 3.2.1. E. Changes in the Daily Routines

This theme covers changes in the everyday routines of the parents and the entire family regarding the family, work, and school setting as well as related challenges and opportunities. The following three subthemes were identified:

(E1) *Challenges regarding the organization and creation of a day structure:* Parents of adolescents with AN faced various changes regarding daily structures and routines, which were challenging for them. These include changes regarding their job situation (working from home, professional reorientation, increased workload), family life (partner and siblings spent more time at home), and social life (fewer social contacts). Additionally, care responsibilities for the child with AN needed to be fulfilled. Parents felt that they needed to create new routines for their children’s lives (e.g., helping with school stuff, keeping the children “occupied”, enabling social contacts with peers): *“They [the children] have needed help with their school stuff […]. Every two weeks, they have got new assignments. Each new assignment was a bit more of work. This was really a challenge. And at noon everyone is hungry. This has to be managed at the same time. And each of us has its own videoconferences […]. That caused me stress”* (#06). In particular, managing the visits of the child in inpatient care was challenging for the one parent who was allowed to enter the hospital building. This was perceived as *“exhausting, travelling to the hospital every day, all the responsibility that you have to bear alone”* (#08).
(E2) Experiencing more time with family: The changed family routines have enabled more family time, which was perceived as positive: “I have had all my children around me; it was very harmonious. We played a lot together, did a lot of things, even prayed, whatever the children wanted to do. Many things were initiated by them. From that point of view, we experienced a lot of positive things as a family out of the fact that we were all together” (#02). More family time has also fostered more intensive conversations between parents and the sick child, between siblings and between the parents, which has “brought us together even more” (#12). For example, one mother described the relationship between the sick child and her siblings as follows: “It’s simply a fact that she [the sick child] has managed to talk with her siblings, especially very intensively with her sister […]. I think this has made a big difference that she has improved so much recently” (#02).
(E3) *Slowing down for the whole family:* COVID-19-related restrictions enabled the whole family *“to slow down”* (#13) and to strengthen self-care behavior. Less school and social stress due to school closures took the pressure off the child diagnosed with AN and provided the opportunity to work on recovery without the fear of missing something, *“an opportunity that can be seized”* (#05). A mother described, *“What is good for her is that the school stress has gone. […] This ‘you have to study now’ and this self-imposed ‘you have to be good at school’ has dropped completely*” (#10).

#### 3.2.2. F. Parents’ Perspective Regarding the Outpatient and Inpatient Treatment

This topic covers the parental perspective regarding the outpatient and inpatient treatment of the children with AN during the COVID-19 confinement. Three subthemes were identified:

(F1) *Reservations about reduced outpatient monitoring and increased teletherapy:* Parents reported that outpatient face-to-face contacts did not take place at all or were significantly reduced, although they acknowledged the clinicians’ effort to keep them going. Nevertheless, face-to-face appointments would have been necessary as *“it is important that they [the patients] are weighed and their urine is checked […] and clinicians see, how they look like, whether there is a relapse or things have improved. This cannot be seen online; this could be totally distorted”* (#02). Remote therapy was seen as an important *“back-up, that she has someone she can talk to […] However, it is, of course, quite different when it takes place face-to-face”* (#10). Providing a place guaranteeing a confidential atmosphere for teletherapy was seen as a basic requirement so that teletherapy can work. Due to the reduced outpatient face-to-face appointments, parents reported to have listened more attentively to any changes in the child’s eating disorder symptoms (*”Of course I was more alert. Otherwise I would have had the feeling that all slips away; and she can tell everything online”* #02) and felt that they should take over the doctors’ responsibility to a certain degree.
(F2) *Altered family contact with the child during the inpatient treatment:* Parents and patients were burdened by the visiting regulations at the ward as the personal contact between the family and the child was strictly limited and “normal” family life was not possible. Particularly, visiting restrictions to the same one parent per week was experienced as burdensome: *“It is difficult for me to understand that it was not possible for the parents to alternate. And for my daughter, this was twice the challenge. I always had the impression that she really suffered from the fact that only one of us could come and that she always had to decide who should come next week”* (#04). A mother described a visit as follows: *“I sometimes felt as if I were visiting her in prison. She also felt like that. She has sometimes stood in front of the windows and said, ‘The people here can go where they want, they can get out, and I′m trapped here.’”* (#08). The short visiting hours and the insufficient availability of space allowing retreat in the visiting area and the obligation to wear face masks during visits were mentioned as burdensome by the parents.
(F3) *Maintaining contact between parents and treatment staff:* Keeping regular contact between parents and clinicians/therapists during COVID-19 confinement to exchange relevant information was regarded as important. While some parents felt well informed, other parents pointed out that they had too little contact with the clinician after therapy has shifted to teletherapy or due to being not allowed to enter the ward during inpatient treatment of the child: *“Sometimes, I would have liked more information as a parent, because I had no contact with the nursing staff at all, because I could not enter the ward. Issues remained unresolved”* (#08). Furthermore, parents emphasized the importance of receiving parental support regarding how to deal with the eating disorder of the child.

#### 3.2.3. G. Challenges and Benefits of the COVID-19 Confinement for the Eating Disorder Symptoms and Mental Health of the Child

Changes in eating disorder and other psychopathology that were observed by the parents during COVID-19 confinement include deteriorations and improvements. The eating behavior was described as increasingly restrictive and selective, on the one hand, which was attributed to fewer social contacts and common family meals that were perceived as irritating and stressful. On the other hand, other parents reported more flexible and regular eating habits, which they also attributed to common family meals and lower social stress: *“She is eating more regularly and faster than in the past. […] This more regular eating is because she is always at home now—during weekdays she had to eat in school together with her friends, and that hasn’t worked out so well. […] And eating together is good for her”* (#10). Regarding physical activity, parents reported difficulties of patients to suppress the urge to exercise (*“I have noticed that she had the urge to walk, walk, walk”* #05). However, most parents described the actual exercise behavior as moderate and not pathological. Regarding mood, some parents described their children as increasingly withdrawn, irritated, and depressive, which was partly explained by not being allowed to leave the hospital building for inpatients and missing daily routines and opportunities to pursue hobbies for outpatients: *“At the beginning, it was difficult for her to deal with the situation, that she was at home all the time, that she could not follow her routines like the dance school, that she could not see her friends at school […] That has set her back. She was more irritated and withdrawn”* (#02). However, other parents described the mood as more cheerful and relaxed, which they attributed to lower social stress and better family relationships.

## 4. Discussion

To the best of our knowledge, this is the first qualitative study focusing on an adolescent population with AN exploring the impact of the COVID-19 confinement. We aimed to explore whether and how the COVID-19 pandemic affected eating disorder symptoms and AN therapy as well as family and school life. In this study, we also included the perspective of the parents as they play a key caregiving role, and their perspectives might differ from the perspectives of their children. Indeed, a general observation from the interviews was that the opinions of adolescents and parents largely coincided, which was true for challenges of the COVID-19 period (e.g., weight monitoring, hospital visiting regulations) as well as for opportunities (e.g., better family relationships, improved self-care). This speaks for the validity of the obtained results.

It must be noted that some of the themes identified in this study might relate not only to adolescents with eating disorders but also to adolescents in general. Particularly, outpatients might have—to some extent—experienced social distancing measures (i.e., homeschooling, exit restrictions) in a similar way as their healthy peers. For example, the theme about feelings of being imprisoned and bored as well as missing close others identified in the present interviews resembles the general observation of increasing social isolation and loneliness among the general population of adolescents during this period [35]. However, as also shown by this study and discussed below, such feelings were linked to reduced motivation to work on recovery and disturbed mood in AN patients. Moreover, improvements in family relationships and more time for relaxation and self-care observed in the present sample were also reported in the general population of adolescents [36,37]. However, as indicated by the results of this study, better family relationships and increased self-care behaviors might be linked to the promotion of recovery in eating disorder patients.

Regarding the impact on the patients’ eating disorder symptoms, the results of this study are consistent with those of a qualitative study including adult patients in the UK, which described the COVID-19 period as a catalyst for either exacerbation of symptoms or recovery [38]. Adolescents and parents reported deteriorations in eating behaviors (e.g., more binge eating, restrictive) and general psychopathology (e.g., elevated levels of anxiety and depression) as well as improvements, including more regular and flexible eating, improved mood, and feeling less stressed. This ambiguity raises the question of potential reasons for either a deterioration or an improvement of symptoms. In this regard, the first aspect to discuss are disruptions in daily routines. Due to homeschooling and exit restrictions, adolescents spent more time at home, which bears the risk of being increasingly exposed to trigger situations. Similar to studies with adult patients [13,24], our study indicates that adolescent inpatients and outpatients experienced more boredom and less distraction, which triggered AN-related cognitions and behaviors. However, disruptions in daily routines may also lead to less everyday stress, which facilitates being more relaxed, as also addressed in a previous study [4]. In our study sample, this was associated with increased self-care behavior, which may have had a positive impact on eating disorder pathology. Another important aspect to consider is the change in family life. As observed in this study, recently published research highlighted increased family conflicts as well as improved family relationships during the COVID-19 period, which has been associated with improvement and worsening of symptoms [4,5,6,39]. This ambiguity may be partly resolved when considering short- and long-term consequences. While more time with the family may have increased family conflicts and irritability at the beginning of confinement, it may have opened up the possibility for joined family activities and improved family relationships in the long term. One specific aspect observed in our study was that parents tended to be more sensitive to changes in the patients’ eating disorder symptoms and to increasingly control their behavior, which may backfire and increase negative feelings in the patients. Similar behaviors were described in caregivers of adult patients [7,24]; however, this risk may be particularly pronounced in adolescents with eating disorders.

The role of school closures in adolescents’ mental health is currently excessively discussed in the public media and scientific literature. While school closures and increased homeschooling are predominantly associated with elevated mental health concerns in adolescents [40,41], the results of this study partly contradict this view. Indeed, adolescent AN patients seem to benefit from school closures as this was related to lower stress levels and having more time to work on recovery. Furthermore, homeschooling may foster self-organization and autonomy skills that are generally considered important by AN patients [42].

Furthermore, changes in the treatment and therapy routine may have had an impact on the course of the eating disorder. Outpatient face-to-face contacts were drastically reduced, which could have only been partly compensated by remote interventions. This is noteworthy because help-seeking behaviors of patients with eating disorders and their caregivers generally increased during the COVID-19 period [43]. Additionally, this study has shown that weight monitoring by patients themselves or their parents bears the risk of reduced outpatient face-to-face contacts. This has not only caused discomfort but also triggered AN-related cognitions towards weight and might have contributed to family conflicts. Finally, changed motivation to work on recovery may negatively influence eating disorder symptomatology during the COVID-19 period, as also brought up in a UK study of adult patients [24]. For inpatients, motivation may be particularly impaired due to restrictive visiting regulations and prohibition to leave the hospital building. Future studies should clarify the reasons that determine whether the pandemic is more of an opportunity or a challenge for eating disorder patients. First studies indicate that differences in emotion regulation and coping skills as well as personality factors may play a crucial mediating role [44,45,46].

### 4.1. Clinical Implications

A key aim of this study was to explore how eating disorder treatment and therapy during the COVID-19 period was perceived and how treatment during this exceptional situation can be improved. Indeed, the findings from this study have implications for clinical practice. This is the first study that sheds light on how COVID-19 restrictions were perceived in adolescent patients receiving inpatient treatment. First, due to exit restrictions and boredom, leisure time at the ward was perceived as less positive. The clinic staff is therefore called upon to offer alternative activities and motivators that are not linked to external contacts that counteract boredom and increase treatment engagement. Second, the clinic management is challenged to find the right balance between strict visiting regulations to prevent COVID-19 infections and patient needs. This study undoubtedly demonstrates that the actual visiting regulations imposed great burden on the adolescent patients and their parents. Being able to see both parents regularly is a basic need of adolescents. Having been forced to decide whether the mother or father is allowed to come to visit was burdensome for patients and meant organizational challenges for parents. Thus, allowing alternate visits from both parents should be considered. Moreover, if visitors are not allowed to enter the patients’ rooms, a visiting area with enough space facilitating appropriate retreat for the family should be provided. Third, in order to ensure smooth treatment continuity for patients, it is important that the communication between clinicians of changing staff cohorts is sufficient and transparent. Rotations of staff cohorts should be reduced to a minimum.

For patients in outpatient treatment, teletherapy was generally well accepted by the patients as it ensures therapy continuity and allows flexibility. However, some basic requirements must be met, including stable Internet connection, use of video instead of audio only, and privacy. These aspects are important for adolescent psychiatry patients in general [21]. The treatment setting and any concerns regarding remote therapy should be evaluated with the patients on a regular basis. For more detailed recommendations on how to perform eating disorder online treatment during the COVID-19 pandemic, we refer to Waller et al. [47] and Matheson et al. [20]. One specific aspect to consider is how the weight monitoring should take place as self-weighing or weighing by parents were perceived as uncomfortable and sometimes boosted eating disorder behavior. This study indicates that regular weight monitoring during face-to-face sessions with the clinicians are important. Alternatively, weighing might be collaboratively performed with the clinician during a video call, as suggested by Murphy et al. [11]. This would also allow for the discussion of any negative feelings related to weighing directly afterwards.

Two further aspects should be considered for both inpatients and outpatients. Therapists should be aware that the motivation to work on recovery might be reduced. Thus, AN therapy during this time should particularly focus on motivational work and goal setting. Furthermore, the present study indicates that communication with parents may have somewhat been neglected. This may be one reason why parents expressed more reservations about teletherapy than patients themselves, a finding that has also been reported in a previous study [24]. Therapists are therefore asked to strengthen communication with the parents of adolescent AN patients during this time (e.g., via video calls), inform them about new interventions (e.g., teletherapy), and discuss difficulties with them. This should ease their fears that their children may not be receiving adequate treatment. Caregiver (online) trainings may be additionally helpful in supporting parents in their caregiving role [48,49]. Parental support should also involve discussions of parents’ responsibilities in caring for their child with an eating disorder considering the additional burden parents face during this time (managing own job, homeschooling of children, household), for example, by emphasizing the importance of self-care.

### 4.2. Limitations

Due to hospital routines and COVID-19 regulations, interviews were conducted face-to-face and online. Moreover, in some online interviews, no bidirectional video connection was possible due to bad Internet connection and WiFi capacity. Different methods may have led to differences in the narrative flow and may not be fully comparable; however, the notes of the interviewers and observers do not provide any indication of this. As emphasized by Weissman et al. [50], traditional methods in eating disorder research may not be fully applied during the COVID-19 period, and some flexibility is needed. One interview with a patient was conducted by a clinician who was directly involved in her therapy. Due the timidity of the patient, who would not have agreed to be interviewed by a person she is not familiar with, this approach had to be taken. As the themes addressed by this patient were in line with the other patients’ interviews and no new themes came up, we decided to keep this interview in the sample. Furthermore, this study included female AN patients only; males and patients with other eating disorder diagnoses (bulimia nervosa, binge eating disorder) were not included. They might have experienced the COVID-19 restrictions differently, as indicated by another study on adult patients [22]. Moreover, the recruited patients were all living in Vienna and its surrounding area. We cannot rule out that patients from other (e.g., more rural) geographical areas would have reported different experiences or opinions. Finally, due to the sample size, we did not separately analyze interviews from inpatients and outpatients. The results from this study indicate that particularly how the COVID-19 pandemic affects treatment is perceived differently among inpatients and outpatients; thus, further research distinguishing the effects on patients in different treatment settings is necessary. However, a strict separation between inpatients and outpatients would not have made sense either because some patients experienced both types of treatment during the COVID-19 period.

## 5. Conclusions

The impact of the COVID-19 confinement on adolescent patients with AN is ambivalent. This period bears challenges and opportunities for the course of the disease, therapy, and family and school life. This study identified some risks of the current treatment practice and overall circumstances, like self-monitored weight or monitoring by parents, reduced motivation to work on recovery, restrictive visiting regulations at the ward, and partially neglected therapist–parent communication. The results provide useful implications on how to improve treatment during the pandemic. On the other side, positive consequences of the COVID-19 period must also be truly acknowledged, which include improved family relationships, being less stressed, and having more time for self-care. This, in fact, raises the question about the pressure society generally places on eating disorder patients and their families and society’s role in the development and maintenance of the eating disorder. Finally, it must be noted that this study focused on patients whose eating disorder started prior to the COVID-19 crisis only. Future studies may focus on adolescents who have newly developed an eating disorder during the COVID-19 period. Our clinical impression indicates that there was an increase in admissions of new-onset AN cases during the last months of 2020 and first months of 2021 with distinct phenomenology and etiology. This may encourage further research regarding the role of COVID-19 confinement in the devolvement of an eating disorder.

## Figures and Tables

**Table 1:** Summary of the main and subthemes identified from the interviews.

Patients’ Interviews	Parents’ Interviews
**A. Restrictions of personal freedom** A1. Feeling of being imprisoned and bored A2. Tensions between patients and family members A3. Less motivation to work on recovery A4. Missing close others **B. Interruption of the treatment routine** B1. Risks through self-monitored weight ^a^ B2. Challenges and opportunities of teletherapy ^a^ B3. Changes of staff cohorts and treatment offers in the inpatient setting ^b^ **C. Changes in the eating disorder and other psychopathology** C1. Boredom and feeling of being observed triggering eating disorder symptoms C2. COVID-19-related fears and compulsions C3. Improvement in symptoms indicating a normal treatment course **D. Opportunities of the COVID-19 period** D1. Less stress allows for paying more attention to own needs D2. Experiencing a more intensive time with the family D3. Promoting autonomy and self-organization skills	**E. Changes in the daily routines** E1. Challenges regarding the organization and creation of day structure E2. Experiencing more time with family E3. Slowing down for the whole family **F. Parents’ perspective regarding the outpatient and inpatient treatment** F1. Reservations about reduced outpatient monitoring and increased teletherapy ^a^ F2. Altered family contact with the child during the inpatient treatment ^b^ F3. Maintaining contact between parents and treatment staff **G. Challenges and benefits of the COVID-19 confinement for the eating disorder symptoms and mental health of the child**

^a^ This theme occurred in outpatients only; ^b^ this theme occurred in inpatients only. All other themes occurred in inpatients and outpatients.

## Data Availability

The data (interview transcripts) are not publicly available as they contain information that could compromise the privacy of the research participants.

## Supplementary Materials

The following are available online at https://www.mdpi.com/article/10.3390/ijerph18084251/s1: Supplementary Material S1. Interview topic guide.
