# Supplementary material for: Impact of COVID-19 Confinement on Adolescent Patients with Anorexia Nervosa: A Qualitative Interview Study Involving Adolescents and Parents
Source: Int J Environ Res Public Health. 2021 Apr 16;18(8):4251. doi: 10.3390/ijerph18084251 (PMC8074137; doi:10.3390/ijerph18084251)
Supplement: Supplementary file 1 [file ijerph-18-04251-s001.zip › ijerph-1131491-supplementary.pdf]

Semi-structured interview - Topic guide

Version Adolescent patients [green]
Version Parents [organge]

[Welcome]

[Explaining the purpose of audio recording and note on confidentiality]: "As we have already described in the information sheet, we will record the interview on audio. We do this because we are not able to write everything down so quickly and in order to be able to transcribe and analyze the interview later on. No one else will hear the audio recording and it will be deleted as soon as the project is finished. The interview will then be anonymized so that your person cannot be identified. You can say everything you are thinking about. We will not tell your doctor, your therapist or your parents / the doctors or therapist of your child what you tell in this interview. Is that okay for you? Dou you have any questions before we start?"

[Start of audio recording]

1. [Introductory question]: "We are interested in the very personal experiences of adolescents / parents of adolescents with eating disorders in connection with the Covid-19 pandemic and the related measures. Maybe you can start to tell how you first heard about Covid-19 and how it went on."

- "What experiences have you had during this time?"
- "What thoughts went through your mind during this time?"

2. [Family life]: "Can you tell me something about family life during that time?"

- "How do you experience the situation with your children?"

3. [School life]: "Can you tell me what the situation with school / education of your child is like during this time?"

4. [Eating disorder]: "Can you tell me something about your illness / the illness of your child during this time?"

- "How is your eating behavior / the eating behavior of your child?"
- "Can you tell me something about your sports behavior / the sports behavior of your child?"

5. [Treatment]: "Can you tell me something about what your therapy or treatment / the therapy or treatment of your child is like right now?"

- "How do you currently experience your treatment and therapy / the treatment and therapy of your child?"
- "What kind of support would you have needed during this time?"

6. [Change due to COVID19]: "Do you think something has changed in your condition / the condition of your child compared to the time before Corona?"

7. [Final Questions]:

- "What – do you think - positive things can you take with you from this current situation?"
- "Is there anything else you would like to tell me?"

[Obtaining Sociodemographic questions]

[Closing questions]:

- "How was this interview for you?"
- "Do you have any further questions?"

[Stop of audio recording, good-bye and thanks for participating in this interview]
